# Supplementary material for: Exploring how and why attributes of existing and emerging early cancer detection tests influence experiences and participation among individuals at risk of socioeconomic disadvantage: A qualitative interview study
Source: PLoS One. 2025 Jul 18;20(7):e0327052. doi: 10.1371/journal.pone.0327052 (PMC12273937; doi:10.1371/journal.pone.0327052)
Supplement: S1 Table — (DOCX) [file pone.0327052.s001.docx]

**S1 Table. Study methods and results reporting according to COnsolidated criteria for REporting Qualitative research (COREQ) checklist**

| **Topic** | **Item** | **Guide Questions/Description** | **Reported in Section  (page no)** |
| --- | --- | --- | --- |
| **Domain 1: Research team and reflexivity** | | | |
| *Personal characteristics* | | | |
| Interviewer/facilitator | 1 | Which author/s conducted the interview or focus group? | Data collection (page 8 - 9) |
| Credentials | 2 | What were the researcher’s credentials? E.g. PhD, MD | Data analysis  (Page 10 -11) |
| Occupation | 3 | What was their occupation at the time of the study? | Data analysis  (Page 10 - 11) |
| Gender | 4 | Was the researcher male or female? | Data collection  (Page 8 - 9) |
| Experience and training | 5 | What experience or training did the researcher have? | Data collection  (Page 8 - 9) |
| *Relationship with participants* | | |  |
| Relationship established | 6 | Was a relationship established prior to study commencement? | Data collection  (Page 8 - 9) |
| Participant knowledge of the interviewer | 7 | What did the participants know about the researcher? e.g. personal goals, reasons for doing the research | Data collection  (Page 8 - 9) |
| Interviewer characteristics | 8 | What characteristics were reported about the inter viewer/facilitator? e.g. Bias, assumptions, reasons and interests in the research topic | Data collection  (Page 8 - 9) |
| **Domain 2: Study design** | | |  |
| *Theoretical framework* | | |  |
| Methodological orientation and Theory | 9 | What methodological orientation was stated to underpin the study? e.g. grounded theory, discourse analysis, ethnography, phenomenology, content analysis | Study design and setting (Page 5) |
| *Participant selection* | | |  |
| Sampling | 10 | How were participants selected? e.g. purposive, convenience, consecutive, snowball | Recruitment and sample (Page 5-6) |
| Method of approach | 11 | How were participants approached? e.g. face-to-face, telephone, mail, email | Recruitment and sample (Page 5-6) |
| Sample size | 12 | How many participants were in the study? | Sample characteristics  (Page 12) |
| Non-participation | 13 | How many people refused to participate or dropped out? Reasons? | Sample characteristics (Page 12) |
| *Setting* | | |  |
| Setting of data collection | 14 | Where was the data collected? e.g. home, clinic, workplace | Data collection (Page 8 - 9) |
| Presence of non-participants | 15 | Was anyone else present besides the participants and researchers? | Data collection (Page 8 - 9) |
| Description of sample | 16 | What are the important characteristics of the sample? e.g. demographic data, date | Data collection (Page 8 - 9) Sample characteristics (Page 12-15) |
| *Data collection* | | |  |
| Interview guide | 17 | Were questions, prompts, guides provided by the authors? Was it pilot tested? | Interview materials (Page 6-8) Public and patient involvement (Page 8) |
| Repeat interviews | 18 | Were repeat inter views carried out? If yes, how many? | n/a |
| Audio/visual recording | 19 | Did the research use audio or visual recording to collect the data? | Data collection (Page 8 - 9) |
| Field notes | 20 | Were field notes made during and/or after the interview or focus group? | Data collection (Page 8 - 9) |
| Duration | 21 | What was the duration of the inter views or focus group? | Sample characteristics (Page 12) |
| Data saturation | 22 | Was data saturation discussed? | Recruitment and sample (Page 5-6) |
| Transcripts returned | 23 | Were transcripts returned to participants for comment and/or correction? | Data analysis (Page 10 - 11) |
| **Domain 3: analysis and findings** | | | |
| *Data analysis* | | | |
| Number of data coders | 24 | How many data coders coded the data? | Data analysis  (Page 10 - 11) |
| Description of the coding tree | 25 | Did authors provide a description of the coding tree? | Thematic framework (Page 15) |
| Derivation of themes | 26 | Were themes identified in advance or derived from the data? | Data analysis (Page 10 - 11) |
| Software | 27 | What software, if applicable, was used to manage the data? | Data analysis (Page 10 - 11) |
| Participant checking | 28 | Did participants provide feedback on the findings? | n/a |
| *Reporting* | | | |
| Quotations presented | 29 | Were participant quotations presented to illustrate the themes/findings? Was each quotation identified? e.g. participant number | Themes (Page 15-22) |
| Data and findings consistent | 30 | Was there consistency between the data presented and the findings? | Themes (Page 15-22) |
| Clarity of major themes | 31 | Were major themes clearly presented in the findings? | Themes (Page 15-22) |
| Clarity of minor themes | 32 | Is there a description of diverse cases or discussion of minor themes? | Themes (Page 15-22) |

A checklist of items that should be included in reports of qualitative research, developed from: Tong A, Sainsbury P, Craig J. Consolidated criteria for reporting qualitative research (COREQ): a 32-item checklist for interviews and focus groups. *International Journal for Quality in Health Care*. 2007. Volume 19, Number 6: pp. 349 – 357
