# Supplementary material for: Exploring how and why attributes of existing and emerging early cancer detection tests influence experiences and participation among individuals at risk of socioeconomic disadvantage: A qualitative interview study
Source: PLoS One. 2025 Jul 18;20(7):e0327052. doi: 10.1371/journal.pone.0327052 (PMC12273937; doi:10.1371/journal.pone.0327052)
Supplement: S2 File — (DOCX) [file pone.0327052.s002.docx]

Understanding public preferences for cancer detection test modalities: a protocol for a qualitative interview study using a vignette ranking task

**Introduction**

Timely uptake of detection tests among those who have cancer affords early diagnosis and curative treatment.[1] However, some populations experience barriers to uptake; cancer screening participation is lower among those with a learning disability,[2] ethnic minority groups[3], those living in areas of high deprivation[4] and those with comorbidities,[5] and later stage diagnosis has been associated with age, gender, income and deprivation.[6] To achieve sustained improvements in early diagnosis, efforts must be directed to improving uptake of tests that detect signs of cancer among eligible individuals at high risk of late diagnosis and cancer mortality.

Physical characteristics of test modalities and the environment in which they are delivered may play an important role in individuals’ capability, opportunity and motivation to take part[7-9] Notably, studies have identified several characteristics of test, often referred to as attributes, that influence preferences and uptake among underserved populations. Changes to the UK bowel cancer screening programme introduced a simpler test requiring one stool sample as opposed to three, increasing engagement by 7% and doubling uptake among those who did not previously participate.[10] Meanwhile, self-sample cervical cancer screening was preferred to clinician sampling by those who had infrequently or never attended screening,[11] and preferences for community-based vs hospital-based lung cancer screening were higher among current smokers and those living in areas of greater deprivation.[12]

Attributes of test modality (such as test setting, procedure, accuracy, person conducting the test etc) are to some extent constrained by the modality itself (type of test) but may be further specified by the healthcare system and cancer diagnosis pathway in which tests are delivered. Consequently, there may be opportunities to accommodate preferences during the design and provision of tests. This lends itself to delivering patient centred care and has been recognised as an important step during the implementation and evaluation of tests for cancer.[13, 14] This approach not only increases the likelihood that the test will be taken up, but also enhances test experience, which together may improve patient outcomes and the likelihood of engaging with tests in the future.[15-17]

Importantly, the cancer detection landscape is rapidly evolving with the development of new types of tests such as multi cancer early detection tests,[18] novel pathways for providing tests to individuals at risk [19, 20], and more sophisticated approaches to risk stratification.[21] The success of implementing these innovations in practice will rely on systematic and simultaneous assessments of public and patient perspectives. This may include comparing preferences for different modalities (e.g. where a new modality may replace an existing test) and evaluating whether preferences for a modality are heterogenous among different populations at risk of cancer (e.g. symptomatic and asymptomatic individuals) and late diagnosis (e.g. those experiencing socioeconomic deprivation). This presents a timely opportunity to understand how cancer detection test modalities can be compared and evaluated across contexts and populations to improve equitable improvements in the uptake of cancer detection tests and patient outcomes.

**Purpose and aims**

A qualitative research design will be used to gather in-depth insights into public perspectives of and trade-offs between the different attributes of cancer test modalities among individuals at higher risk of late diagnosis. The aim of this study is not to generate recommendations for designing or delivering specific detection tests, but to more broadly conceptualise the attributes of tests that are important to define and explore when evaluating public and patient preferences for a given test modality in different contexts and populations. This will facilitate dynamic and holistic approaches to considering and comparing preferences across modalities and cancer pathways in support of achieving equitable and patient-centred improvements in early diagnosis.

**Objectives**

To conceptualise and define the diverse range of attributes of cancer detection test modalities important for participation, from the perspective of individuals experiencing socioeconomic deprivation who are at risk of late diagnosis

To understand preferences for different attributes of test modalities among those experiencing socioeconomic deprivation, by observing how (and why) participants make tradeoffs between these test attributes in different hypothetical contexts

**Methods**

*Design*

A qualitative interview study using semi-structured interview questions, fictional vignettes and an adapted think-aloud protocol, to encourage and observe the cognitive processes of deliberation and trade-off between different attributes of cancer detection tests that are determined and influenced by test modality.

*Procedure*

Participants will first be asked about their previous experiences of being offered and engaging with cancer detection tests. This will allow participants to comment openly on the attributes of tests they have had or are aware of that they feel are important for acceptability and uptake. A topic guide will be used to ask participants about their opinions, however the nature and length of the discussion will depend on individual experiences.

Next, two separate vignette ranking tasks will be presented to participants. Before engaging with these tasks, participants will be given a brief description of the different types of test modality (imaging, sample, endoscopy and biopsy) to ensure that those who have no previous experience with cancer tests understand what they might involve.

Each vignette task will introduce a fictional individual who has been identified as being at high risk for cancer. One individual will represent those eligible for national screening programmes and will be described as experiencing no symptoms but belonging to a high risk demographic group. The other will represent individuals experiencing symptoms that may indicate cancer, representing those who might be offered a detection test after presenting to their local GP or A&E department.

In each task, participants will be presented with descriptions of four different hypothetical cancer detection tests and asked to rank them in the order in which they think the individual at risk would prefer them. During each exercise, participants will be asked to express their thought processes, feelings and reasons for their decisions out loud, using a concurrent think-aloud protocol. Participants’ interpretation and comprehension of the different attributes will also be observed. Questions and prompts (see Topic guide) will be used flexibly to ask participants about the reasons for the chosen order and to discuss attributes in more detail. Participants will be free to change the order of the vignettes throughout the discussion. They will be invited to take part in a practice task to familiarize themselves with the think-aloud approach beforehand.

The four hypothetical test scenarios within each task will be presented simultaneously in a randomly shuffled order to each participant to prevent order effects.[22] The order of the two tasks will also be varied by participant. Discussions will be guided by participants’ reasons for their decision order and their attentional focus. If participants find the task difficult, they will be encouraged to focus on two tests of their choice and discuss those freely. They may also be asked to select the most and least preferred test, leaving the other two tests placed equally, akin to diamond ranking, a research tool found to facilitate the ordering of difficult items.[23] In other situations, participants who have completed both tasks may be invited to compare all 8 tests to one another to prompt further considerations of the attributes.

To ensure acceptability and comprehension of the task, as well as to improve face validity, the vignettes and topic guide will be reviewed by two public and patient representatives and an academic with expertise using vignettes in qualitative research.

*Vignette design*

Key attributes of cancer detection test modalities were mapped out using an evidence synthesis exercise. The aims were to include as many of these attributes in the vignettes as possible and where an attribute had multiple levels, to use maximum variation as a comparison. Each vignette contained 3-5 attributes which were presented chronologically wherever possible in terms of procedure (e.g. duration) and outcome (e.g. results time).

*Sample*

Thirty individuals aged 50 years and older will be sampled purposively using an area level marker of deprivation (those living in top 30% most deprived areas of England as measured by index of Multiple deprivation, 2019[24]) and an individual level of marker (semi-routine and routine manual and service occupation category according to the National Statistics Socio-economic classification 2020,[25] low income – thresholds defined as minimum household incomed for acceptable standard of living in the UK, determined by household and retirement status (£16,996 – single pensioner, £26,113 – two pensioners, £24,876 - single person working/unemployed, £34,494 – couple working/unemployed)[26] and/or social rent housing tenure[27]). Individuals with a previous cancer diagnosis in the last 5 years will be excluded.

In order to further characterise the sample, participants will be asked to report their gender,[28] education, ethnicity,[29] and previous test experience and will be asked questions measuring general health status,[30] and health literacy.[31] This information will be used to monitor the sample on an ongoing basis in order to ensure maximum diversity wherever possible, but the primary focus will be on including those with the above specified markers of deprivation.

*Recruitment*

​Participants will be recruited via proactive community-based methods. Community champions and grass roots organisations (such as social housing associations, community groups, local councils, public health authorities, and large employers in manual occupation industries) will be approached in areas associated with high deprivation in London to assist in recruiting individuals meeting the inclusion criteria. These organisations will be contacted in the first instance to establish how best to engage and reach individuals from deprived communities and to ensure that the approach used is appropriate and sensitive to the population.

​Individuals will be recruited directly by the researcher or via the community gatekeeper depending on the circumstances and what is deemed most appropriate by the community organisations. Individuals will be recruited opportunistically in person at locations recommended by community organisations or remotely via adverts and social media platforms (e.g. poster, email, online newsletter, social media advert etc). Where individuals are not recruited directly by the researcher, the researcher’s contact details will be provided so that they can express their interest.

​

*​Interviews*

​Individuals will be invited to take part in a one to one semi-structured interview (up to 60 minutes). Given that vignette based tasks are easier to conduct in person,[22] the interviews will be carried out face to face wherever possible. However, if upon expressing interest in the study it is clear that this will be inconvenient or difficult for an individual meeting the inclusion criteria, the interview will be set up via an online video-calling platform (e.g. Microsoft Teams, WhatsApp). In this scenario, the vignette tasks will either be shared on screen or posted to participants in advance of the interview.

The vignettes and topic guide will be piloted with a public and patient representative prior to conducting the interviews to ensure that the task is easy to understand and feasible for participants to engage with.

​

*​Data collection*

​The final order of the vignettes will be recorded for each participant. This will provide a descriptive measure of differences and similarities across the sample. However, the purpose of the vignette task is to draw attention to the attributes of tests and various trade-offs between them that are important for preferences and thus the qualitative discussions will be the focus of analysis. All interviews will be audio recorded using an encrypted Dictaphone and transcribed verbatim by the researcher or a professional transcription software/company (with appropriate data processing agreements in place) where personally identifiable data will be omitted during transcription or before data analysis. Recordings will be deleted once each transcript is complete.

*Analysis*

​The qualitative data will be analysed using applied thematic analysis in NVivo software. A pragmatic approach will be taken to analysing the data. Initially a few transcripts will be coded inductively by at least two members of the research team. The research team will then meet to discuss and refine a framework for analysing the remaining transcripts. The final framework may be loosely organised by the following categories: attribute definitions, attribute trade-offs, interactions between attribute preferences and contextual and population factors (e.g. symptomatic vs asymptomatic).

*Costs*

​In line with NIHR guidance, participants will receive £25 for taking part in the study in recognition of their time and any costs associated with taking part in the interview (travel costs, internet use). This will be provided in the form of vouchers.*​*

References

1. Crosby, D., et al., *A roadmap for the early detection and diagnosis of cancer.* Lancet Oncol, 2020. **21**(11): p. 1397-1399.

2. Osborn, D.P.J., et al., *Access to Cancer Screening in People with Learning Disabilities in the UK: Cohort Study in the Health Improvement Network, a Primary Care Research Database.* PLOS ONE, 2012. **7**(8).

3. Halpern, M.T., et al., *Association of insurance status and ethnicity with cancer stage at diagnosis for 12 cancer sites: a retrospective analysis.* Lancet Oncol, 2008. **9**(3): p. 222-31.

4. von Wagner, C., et al., *Inequalities in participation in an organized national colorectal cancer screening programme: results from the first 2.6 million invitations in England.* International Journal of Epidemiology, 2011. **40**(3): p. 712-718.

5. Diaz, A., et al., *Association between comorbidity and participation in breast and cervical cancer screening: A systematic review and meta-analysis.* Cancer Epidemiology, 2017. **47**: p. 7-19.

6. Cancer Research UK. *Cancer mortality statistics*. [cited 2023 June]; Available from: <https://www.cancerresearchuk.org/health-professional/cancer-statistics/mortality#heading-Three>.

7. Michie, S., M.M. van Stralen, and R. West, *The behaviour change wheel: A new method for characterising and designing behaviour change interventions.* Implementation Science, 2011. **6**(1): p. 42.

8. Robb, K.A., *The integrated screening action model (I-SAM): A theory-based approach to inform intervention development.* Preventive Medicine Reports, 2021. **23**.

9. Marteau, T.M., G.J. Hollands, and P.C. Fletcher, *Changing human behavior to prevent disease: the importance of targeting automatic processes.* science, 2012. **337**(6101): p. 1492-1495.

10. Moss, S., et al., *Increased uptake and improved outcomes of bowel cancer screening with a faecal immunochemical test: results from a pilot study within the national screening programme in England.* Gut, 2017. **66**(9): p. 1631-1644.

11. Drysdale, H., et al., *Self-sampling for cervical screening offered at the point of invitation: A cross-sectional study of preferences in England.* Journal of Medical Screening, 2022.

12. Balata, H., et al., *Attendees of Manchester’s Lung Health Check pilot express a preference for community-based lung cancer screening.* Thorax, 2019. **74**(12): p. 1176-1178.

13. Walter, F.M., et al., *Evaluating diagnostic strategies for early detection of cancer: the CanTest framework.* BMC Cancer, 2019. **19**(1): p. 586.

14. Handley, M.A., A. Gorukanti, and A. Cattamanchi, *Strategies for implementing implementation science: a methodological overview.* Emergency Medicine Journal, 2016. **33**(9): p. 660-664.

15. Gooberman-Hill, R., *Qualitative Approaches to Understanding Patient Preferences.* The Patient: Patient-Centered Outcomes Research, 2012. **5**(4): p. 215-223.

16. Ouellette, J.A. and W. Wood, *Habit and intention in everyday life: The multiple processes by which past behavior predicts future behavior.* Psychological bulletin, 1998. **124**(1): p. 54.

17. Williams, D.M. and D.R. Evans, *Current Emotion Research in Health Behavior Science.* Emotion Review, 2014. **6**(3): p. 277-287.

18. Cancer Research UK. *The Galleri multi-cancer blood test: What you need to know*. 2021 [cited 2023 March]; Available from: <https://news.cancerresearchuk.org/2021/09/13/the-galleri-multi-cancer-blood-test-what-you-need-to-know/#:~:text=A%20blood%20test%20that%20has,cancer%20cells%20into%20the%20blood>.

19. Erridge, S., et al., *Rapid Diagnostic Centres and early cancer diagnosis.* British Journal of General Practice, 2021. **71**(712): p. 487-488.

20. Harris, M. *UK NSC recommends introduction of targeted lung cancer screening*. 2022 [cited 2023 April]; Available from: <https://nationalscreening.blog.gov.uk/2022/09/29/uk-nsc-recommends-introduction-of-targeted-lung-cancer-screening/>.

21. Loomans-Kropp, H.A. and A. Umar, *Cancer prevention and screening: the next step in the era of precision medicine.* npj Precision Oncology, 2019. **3**(1): p. 3.

22. Steiner, P.M., C. Atzmüller, and D. Su, *Designing Valid and Reliable Vignette Experiments for Survey Research: A Case Study on the Fair Gender Income Gap.* 2017, 2017. **7**(2): p. 43.

23. Clark, J., *Using diamond ranking as visual cues to engage young people in the research process.* Qualitative Research Journal, 2012. **12**(2): p. 222-237.

24. UK Government. *The English Indices of Deprivation 2019 (IoD2019)*. 2019 [cited 2023 March 2023]; Available from: <https://assets.publishing.service.gov.uk/government/uploads/system/uploads/attachment_data/file/835115/IoD2019_Statistical_Release.pdf>.

25. Office for National Statistics. *SOC 2020 Volume 3: the National Statistics Socio-economic Classification (NS-SEC rebased on the SOC 2020)*. 2020 January 2023]; Available from: <https://www.ons.gov.uk/methodology/classificationsandstandards/standardoccupationalclassificationsoc/soc2020/soc2020volume3thenationalstatisticssocioeconomicclassificationnssecrebasedonthesoc2020#deriving-the-ns-sec-full-reduced-and-simplified-methods>.

26. Joseph Rowntree Foundation. *A minimum income standard for the UK in 2022*. 2022 [cited 2022 September 2022]; Available from: <https://www.jrf.org.uk/report/minimum-income-standard-uk-2022>.

27. Office for National Statistics. *Housing Tenure by Borough*. 2020 January 2023]; Available from: <https://data.london.gov.uk/dataset/housing-tenure-borough>.

28. EDIS. *DIVERSITY AND INCLUSION SURVEY (DAISY) QUESTION*

*GUIDANCE - WORKING DRAFT (V2)*. 2022 [cited 2024 January]; Available from: <https://edisgroup.org/wp-content/uploads/2022/05/DAISY-guidance-current-upated-May-2022-V2.pdf>.

29. Office for National Statistics. *Ethnic group classifications: Census 2021*. 2021 [cited 2024 February]; Available from: <https://www.ons.gov.uk/census/census2021dictionary/variablesbytopic/ethnicgroupnationalidentitylanguageandreligionvariablescensus2021/ethnicgroup/classifications>.

30. DeSalvo, K.B., et al., *Predicting Mortality and Healthcare Utilization with a Single Question.* Health Services Research, 2005. **40**(4): p. 1234-1246.

31. Morris, N.S., et al., *The Single Item Literacy Screener: Evaluation of a brief instrument to identify limited reading ability.* BMC Family Practice, 2006. **7**(1): p. 21.
