# Supplementary material for: Exploring how and why attributes of existing and emerging early cancer detection tests influence experiences and participation among individuals at risk of socioeconomic disadvantage: A qualitative interview study
Source: PLoS One. 2025 Jul 18;20(7):e0327052. doi: 10.1371/journal.pone.0327052 (PMC12273937; doi:10.1371/journal.pone.0327052)
Supplement: S3 File — (DOCX) [file pone.0327052.s003.docx]

### S3 File. Interview materials

The interview topic guide including questions and prompts, as well as the interview tools (test examples and vignette tasks) are shown below:

**Interview topic guide**

*Note, the questions are designed to serve as a topic guide. They are not definitive and may be used flexibly or adapted in response to information provided by the participant. Not all participants will be asked all questions – it will depend on each participant’s individual experiences and time constraints based on topics participants would like to discuss.*

Testing experience

1. Have you ever been invited to have a cancer test?  
   *PROMPT*: Have you been invited to a cancer screening programme – bowel, cervical, breast? 
   *PROMPT*: Have you ever visited/thought about visiting your doctor for symptoms that might have indicated cancer?
   *PROMPT*: What was the test and do you know why it was offered to you?

*Researcher note: Ask questions 2-8 (if applicable) for each type of test mentioned in question 1 
**Researcher note: If participant has never been invited for a test, skip to question 11 

1. Do you remember what you considered/how you felt when deciding whether or not to take the test? 
   *PROMPT*: Did you think about how easy it would be for you to get to the test? 
   *PROMPT*: Did you think about what the test would involve? 
   *PROMPT*: Did you think about what the test results could tell you and what they might mean?

*PROMPT*: What did you think the potential benefits or risks of having the test would be?

1. Did you decide to go for the test?

** Researcher note: If answer is no skip to question 9

1. What made you want to have the test?
2. What was your experience of being able to get the test? 
   *PROMPT*: Where and when could you have the test? 
   *PROMPT*: Did you have to travel? 
   *PROMPT*: Was it easy or difficult?
3. What was your experience of having the test?

PROMPT: Did you understand what you needed to do?
*PROMPT*: Was it comfortable or uncomfortable? 
*PROMPT*: Did it take long? 
PROMPT: Were there any disadvantages to you?

1. What was your experience of receiving the results?  
   *PROMPT*: Did you get them straight away or did you have to wait?

*PROMPT:* How did you receive them?
*PROPMPT:* What did it mean you had to do next?

1. What would have improved your experience of getting tested? 
   *PROMPT*: Was there anything that you found off-putting? 
   *PROMPT*: Is there anything you would warn someone else about if they were going for the test? 
   PROMPT: Would you want anything to be done differently if you had the test again?

No/limited previous testing experience

Participant was offered a test/thought they should go to their doctor for a test, but did not take a test/visit doctor:

1. Can you tell me about any times where you have been invited to or offered a test but didn’t get round to going or decided not to?
   PROMPT: What was the test?
   PROMPT: Do you know why you were offered it?
2. Were there any reasons why you didn’t get round to it/ why you decided not to get tested? 
   *PROMPT*: How easy/difficult would it have been to have the test?
   *PROMPT*: What did you think having the test would involve? 
   *PROMPT*: Would having the test have disadvantaged you in any way?
   *PROMPT*: Did you think about what the results would tell you?

Participant has never been invited for a test:  

1. If you were invited to have a test for cancer, what do you think would be important to you in deciding whether to have it or not?
   *PROMPT*: type of test/where/when/how/who/why?  
   *PROMPT*: A particular circumstance – symptoms/location/time 
   *PROMPT*: Is there anything about having the test that would be inconvenient/ off putting?

PROMPT: Would there have been any advantages or disadvantages?

**Test examples**

[Instructions]

I will now show you some descriptions of the types of test that can be used to detect signs of cancer. These are not always used to look for cancer, and may be used to look for many other diseases and health conditions. A patient may need more than one test, and in some case a combination of tests may be used. Please read through these in your own time.


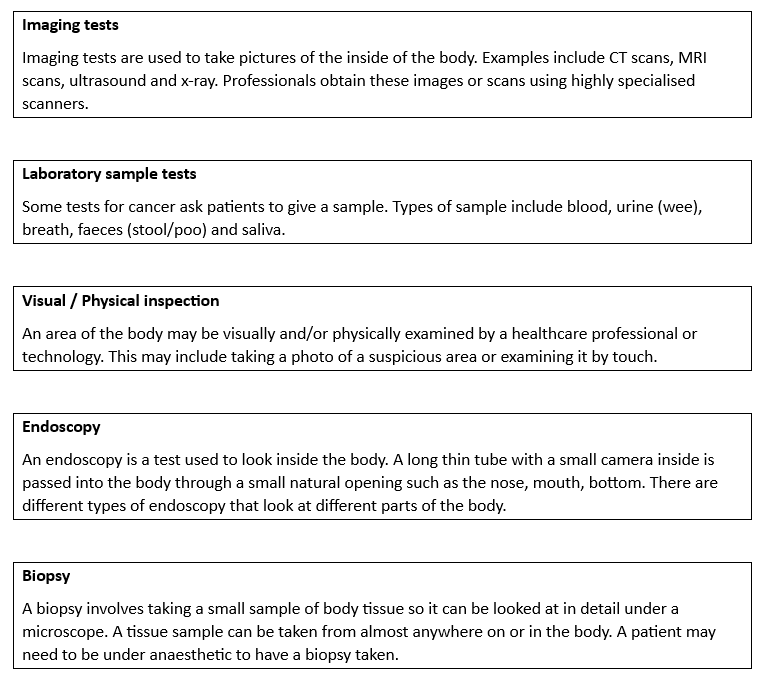


**Figure 1.** Interview materials: Test modality examples

1. *Do you have any first impressions or thoughts on these tests?*
   *PROMPT: What do you think would be encouraging/off-putting about them to patients?*
   *PROMPT: Is there anything about them a patient would want to know before having the test?*

**Vignette task**

[Instructions]

Now I want to tell you about some fictional individuals and hypothetical/made-up tests for cancer that they might be offered. I would like you to imagine what the individual would think about the tests if they were asked to choose between them. This is not a test and there are no correct answers. Everyone has different opinions and I am just interested in finding out what people think about the different options. I want you to be comfortable doing this activity, so please let me know if you feel uncomfortable and would like to stop at any point.

Please read the description of the individual and then read the different test descriptions while “thinking aloud”. This means that I would like you to look over the materials and while you do so, say what you are thinking and feeling and what you think a patient’s initial reaction and first impressions of these tests might be. You may read the text out loud if you would like to, but there is no need to do so if you’d prefer not to.

Please be honest while you are thinking aloud, there’s no need to be polite. It’s really helpful to know what people’s first impressions of these tests would be. Thinking aloud usually feels a bit strange at first, as it is an unusual thing to do. We can do a practice exercise first so that you can get used to it.

Jack has to go into work next week and needs to take public transport to get there. Which option do you think he would prefer?

Option A: The bus will take 1 hour and is likely to be busy. The bus stop is near to his house.

Option B: The train will take half an hour and is more expensive than the bus.

**Figure 2**. Vignette practice task


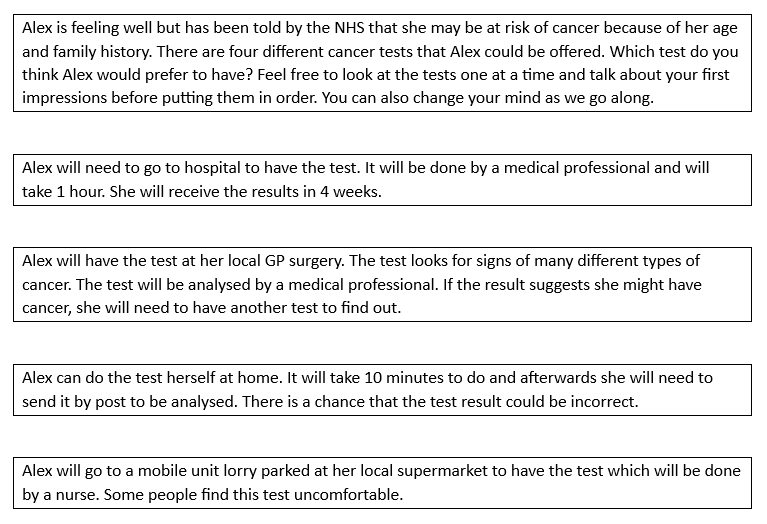


**Figure 3.** Interview materials: Asymptomatic vignette task and test options


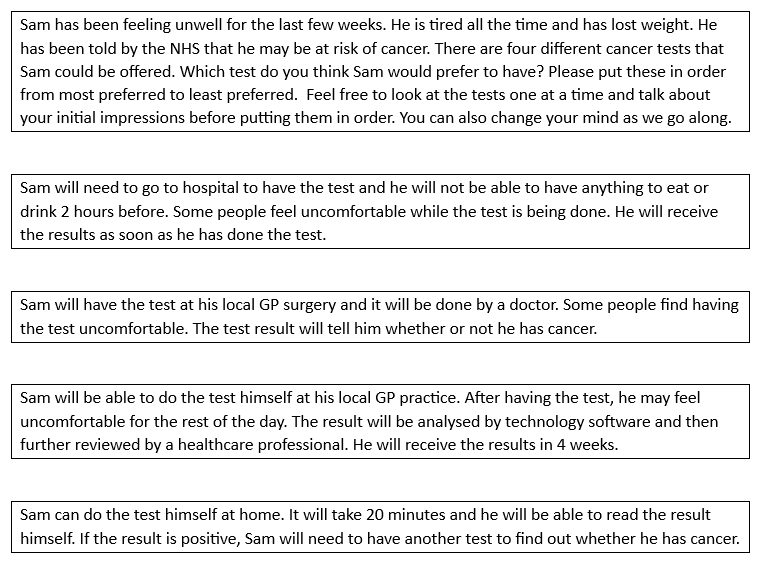


**Figure 4.** Interview materials: Symptomatic vignette task and test options

*The following questions, prompts and probes will be used flexibly to initiate and/or guide discussions during the think aloud exercises and after participants have put the tests in order. Participants may also be asked more flexibly to elaborate on things they say during the task, and be provided with examples of alternatives to the attributes mentioned in the scenarios.*

1. How easy or difficult was it to decide between the different scenarios?
   *PROMPT*: I noticed that you spent some time deciding between x and y, what were you thinking about?
   *PROMPT*: You seemed to decide quite quickly between x and y, was that an easy decision?
2. What do you think about Test X/characteristic X would be appealing or inconvenient?
   PROMPT: What would make it easier/more convenient?
   PROMPT: Would some people find it easier/more inconvenient than others? Why?
3. What made you decide to put these two vignettes in this order?
   *PROMPT*: What about that one, why do you think Sam/Alex would prefer that?
   *PROMPT:* Why do you think these two vignettes are equal to one another?
   *PROMPT*: Why is this one better than that one?
   *PROMPT*: Why have you put that vignette first/second/third/last?
4. What aspects of the tests do you think would be most important when making the decision?
5. When you were deciding between them, was there anything else that you wanted to know about what the test would involve?
6. Do you think everyone would put these in the same order?
   *PROMPT*: Can you think of anyone you know who might have a different view?
   PROMPT: Would it depend on an individual’s circumstances?
   *PROMPT*: Can you think of any reasons why people might order them differently?
7. Are there any circumstances under which an attribute would be more/less important?
   *PROMPT*: Would it matter why Sam/Alex was offered the test?
   *PROMPT*: What about whether they felt well/unwell, how long they had been waiting for the appointment, whether they had had the test before?
8. Looking at all the tests, what do you think the ideal combination would be?
   *PROMPT*: Which characteristics would you put together?
   *PROMPT*: Would you adapt any if it was possible?
