# Supplementary material for: Exploring how and why attributes of existing and emerging early cancer detection tests influence experiences and participation among individuals at risk of socioeconomic disadvantage: A qualitative interview study
Source: PLoS One. 2025 Jul 18;20(7):e0327052. doi: 10.1371/journal.pone.0327052 (PMC12273937; doi:10.1371/journal.pone.0327052)
Supplement: S4 Table — (DOCX) [file pone.0327052.s004.docx]

**S4 Table. Illustrative quotes**

| Theme and sub-themes | Illustrative quotes |
| --- | --- |
| **Theme 1: Attributes can prevent equitable access to cancer detection tests** | |
| Required time commitments | *“I was looking after my mum who was bedridden, also my little boy's special needs.... Even though he's 12, I can't really leave him very much on his own because he is autistic… I'm a single parent, I've got no family over here …. and I can't go just pay a babysitter £20, it's just not possible on part time wages and a single parent with the cost of living”* Female, age 50-59, Mixed  *“They might lose work, have to catch up on work. They might not be able to get out of work.”* Male, age 50-59, White  *“Don't you think it's a little bit off putting about a hospital? It's like, pfft, I could be here all day….my local GP's five minutes walk. Instead of going to the hospital….”* Male, age 50-59, White  *“The test might take an hour, but sometimes you're sitting in a hospital waiting room and you could be waiting a lot longer for an hour and then you've got to get there so maybe a lorry in a local supermarket would be better,”* Female, age 50-59, White |
| Accessibility of the test location and environment | *“Yeah, that seems reasonable. Because it's local.”* Female, age 60-69, White  “*One of them was right in the middle of <London borough> and it's absolutely hopeless to park and then you spend half the time roaming the streets looking for a place to park so*” Female, age 70 - 79, Mixed  *“I didn't, haven't been --Which is naughty, but I don't know, it seemed a lot more effort to get there than it used to because I can walk to a closer hospital in 10 minutes.”* Female, age 62, White  *“They have quite high metal steps normally if it's like the cabin thing and they're not easy to get up and get down again now, yeah. …. Yeah. I don't fancy the mobile unit and a lot of them wouldn't like the stairs ….. Yeah, because some of my friends they find it difficult getting on and off the buses.”* Female, age 70-79, White  *“I am diagnosed autistic and I have Asperger's syndrome and one of the things that affects me is uncontrolled loud noise. So if I'm sitting in a public space, where you've got people chatting away on their mobile phones, you've got a baby screaming, television blaring out, someone playing games on their phone, my brain can't filter that”* Male, age 50-59, White  *“The X Hospital there is a rather cold setting. It's not very, it's very standard, it's not a nice little place or anything like that…. but the place could look, could be made more cosier.”* Female, age 60-69, Mixed  *“They are uncomfortable, them units….. Not the test but the environment …., but the space, it's a little bit on the cramped side.”* Female, age 70-79, White |
| Stigma, privacy and support | [discussing testing in a mobile unit parked in a supermarket] *“People's lining up and you might see someone that you don't want them seeing you or thinking, oh, he's gone for a cancer scare or something.”* Female, age 70-79, White  *“Some people are very negative on, when they are examining you. And it's sort of like, you know, we can't all walk in here a size 10, ….. So it's that side of things, I think as a woman.”* Female, age 60-69, White  *“Because it's easy, you can do it in your own, in the privacy of your own home.”* Female, age 60-69, White  *“For me it was somewhat embarrassing to have this breast out and my clothes off and stuff like that,”* Female, age 60-69, Mixed ethnicity  *“When it was a case of the prostate, it was a woman doctor, a young doctor and she decide to look my, private part we call it, you see? And I feel a bit uncomfortable because was a lady and much younger at me.”* Male, age 70-79, Mixed  *“There's no way I would test myself for any cancer if I didn't have friends and family around to support me.”* Age 60 – 69  *“Because I think people, when they're getting opinions of the health care professionals, it puts them more at ease as well.”* Male, age 50-59, Black  *“because of the way they literally use you, I feel, in my mind, like a slab of meat. They're not very compassionate when doing mammograms… when they put that machine down on you, it's so hard”* Female, age 60-69, Mixed  *“It's really helpful if they're nice and caring and understanding, because I don't like needles, so they can really help if they are caring and understanding,”* Female, age 60-69, Mixed |
| Confidence to take part (self-efficacy) | *“I think that's pretty standard, isn't it? This feels like a normal part of a doctor's appointment. I feel that would be fine. I don't think there's anything unusual or unexpected about that.”* Male, 50-59, White  *“A lot of my friends would feel happier going to the local surgery. It's easier for them and they probably know the staff and yeah, so that would probably be very popular.”* Female, age 70-79, White  *[bowel screening] “Because it was the first time, that's why I was hesitant. Otherwise, I wouldn't be.”* Female, age 50-59, Black  *[discussing cervical cancer screening] “I've never had a baby, but I imagine perhaps if you've had children, you're more used to that sort of thing, but to me, it's very invasive.”* Female, age 60-69, White  *“I think the idea that you're going to ask patients to test themselves for cancer to me is just putting way too much responsibility on patients.”* Age 60 -69  “*Personally, you do the test yourself, it will be in your mind, "have I done it right?" … You don't want to misdiagnosis because you've done something wrong, yeah? That could be killing yourself really, couldn't it?”* Male, age 60-69, White  *“I think tests at home, they're usually quite simple ones…Anything that's easy to do, I think most people wouldn't mind.”* Female, age 50-59, Asian  *“Especially if you're not mobile enough to get out of your house, how are you going to do your testing at home safely and accurately?”* Male, 50-59, White  *“I would say, do you want it to be done while you're awake or while you're asleep? And then I could be given the choice.”* Female, age 50-59, Black  *“She didn't let me know that there was a smaller option. There's different sizes that you can use, and so she should have done because I was in great pain … If they was going to do the tests, yeah, I think, like I was saying, it would be helpful to, for the patients to understand what their options are.”* Female, age 60-69, Mixed |
| Physical and psychological discomfort | *“Couple of days, I have to take painkillers because they've been squashed so much. And I know, talking to other people, they feel the, other women, they feel the same, it's something that people, I know really dread.”* Female, age 60-69, White  *“Because a friend of mine went for a biopsy, and according to him, it messed him up ….. and he walks funny now. So that one there, I've always been a bit sceptical of that because of that instance there”* Male, age 50-59, Black  [cervical screening*] “I didn't mind so much when I was younger, it wasn't a problem, it's, it is quite an invasive and very intimate thing to do but as you get older, I've gone through the menopause and it's painful….”* Female, age 50-59, Mixed  *“I don't want to go back, because my friend also said that it's painful and then she doesn't want to go any more.”* Female, age 50-59, Asian  *“I wouldn't have a biopsy without going on the proper, I would do it as an operation, not just standing there, like being slaughtered.”* Female, age 50-59, Black  *“You don't know what that person has been through in the past. They might have had a lot of traumatic incidents … then if you're going to get stuck in the tunnel for the MRI scan, it's giving me flashbacks of being confined and scared–”* Female, age 50-59, Mixed |
| **Theme 2: Attributes are associated with the perceived risk, harms and benefits of cancer testing and diagnosis** | |
| Attributes associated with perceived risk of a cancer diagnosis | *“When you get the faeces sample, you do that as a general one.”* Female, age 60 -69, White  [GP practice] “…*probably might be less worrying than going straight to the hospital, that could be a worry, it's a different environment and a bit more scary that there's something wrong with you.”* Female, age 70-79, White  *“The fact that the test is done by a doctor rather than say a nurse or a nurse practitioner suggests it's a bit more serious*.” Male, 50-59, White  *“When I had the bubble thing [colonoscopy], it was in the last year…. an offer of the kit was sent out in that same period as well, and I thought, well, no, I'm not going to do it double.”* Female, age 60 -69, White ethnicity  *“Bowel cancer test kit came in the post. I didn’t ask for it, I don't know why it came. … my half-sister’s had bowel cancer ….. just because she's had it doesn't mean I'm going to have it… I just didn't feel I wanted to engage with that…. I think I more or less threw it away.”* Age 60-69 |
| Attributes associated with harms of waiting for or receiving a cancer result | *“nothing worse than that, hanging on and hanging on.”* Female, age 70-79, White  *“I was told I had kidney cancer …..it was a terrible time, I thought I was dying. Then I went back to the hospital and they apologised, they gave me the wrong person's result…. I nearly gave myself kidney cancer, I think I had, the amount of alcohol I drunk.”* Male, age 60-69, White  *"But I can just remember back, it was genuinely the worst night of my life, because obviously your head goes to the cancer straight away.”* Male, age 56, White ethnicity  *“I wouldn't want to know if I had got cancer, because then I'd have to tell my family, and I don't know whether I could do that. Not right now while my mum's still alive, because she's got her own health issues and stuff …. she'd just get too upset.”* Female, age 50-59, White |
| Attributes associated with benefits of an accurate or quick cancer result | *“When you hear the big C …. I'd prefer to have a test done in a medical environment”.* Female, age 70-79, Mixed  *“especially like a, something like cancer, you just, I do take it straight to the professionals.”* Male, age 50-59, Black  “*If you feel uncomfortable when the test is being done, if it's, if we're talking about cancer, then I guess you put up with it, don't you?”* Female, age 60-69, White  *“If you did it at the local GP perhaps at least you're moving along the line, aren't you? And then at least when you get the result, four weeks is quite reasonable, isn't it? And you're going to get a proper result”* Female, age 70-79, White  *“I wouldn't particularly feel comfortable with the breath one …. I don't think that's strong enough to give me a good, strong result”* Female, age 60-69, Mixed  *“I have read in some people, when they've had the breast things done …they've said, oh, there's something, and then there hasn't been, and they've gone and had a procedure, or a biopsy for nothing..”* Female, age 60-69, White ethnicity  *“If you've got a worry you've got cancer you want to know straight away so you can move forward with it”* Female, age 50-59, White  *“If I had to wait six weeks to go to the hospital, which I would prefer, I'd rather get going and do the worst one really, make a start.”* Female, age 70 - 79, White |
| Preferences for informed choice about cancer testing | *“So I think with things like that, the imaging, it's, what I find really unsettling is, you go there, you don't know what they're looking at”* Female, age 60-69, White ethnicity  *“I think the last thing you'd want is to feel that you gave some sample of whatever kind and the next thing you are being rung to say you've got cancer” Age 60-69*  *“The information that I got … it was confusing. They didn't explain everything to me properly. I wasn't, at the initial outset, they didn't tell me the risks of having the cancer test, of having the test. Yeah. So they told me later that if they put the camera inside that it could be fatal.”* Male, 64, White  *“Full information, that is what I believe patients should have. They need all the information so they can make a fully informed choice, rather than being told, just have it and then you find out afterwards, oh my God, maybe I didn't want to have it.*” Female, age 60-69, White  *“I'd like as much information about the test that I've been offered as possible prior to it so I could make up my mind whether I wanted to have it or not….. I'd really like to know all the steps involved in terms of what the test is, how long it will take, when I'd get the results and all that information, I'd like that prior…. I'd like to know, well, basically why, if it's, is my particular demographic more susceptible to a particular type of cancer and that's why I'm being offered the test? Is it a mass screening?”* Female, age 70-79, Mixed |
