# Supplementary material for: Exploring how and why attributes of existing and emerging early cancer detection tests influence experiences and participation among individuals at risk of socioeconomic disadvantage: A qualitative interview study
Source: PLoS One. 2025 Jul 18;20(7):e0327052. doi: 10.1371/journal.pone.0327052 (PMC12273937; doi:10.1371/journal.pone.0327052)
Supplement: Attachment — Submitted filename: Response to reviewers 15.05.25.docx [file pone.0327052.s006.docx]

Dear Veincent Christian Pepito,

We are very grateful to you and the reviewers for taking the time to review the paper and provide meaningful feedback. We have addressed the journal requirements including updating the file labels, manuscript format and data availability statement. We have also changed the reference numbered 45 (a report published by Cancer Research UK) to the more recently published and associated journal article. The reference numbered 48 has been updated to an alternative publication as the previous article had been redacted.

We have now addressed each comment from the reviewers below and believe these revisions have significantly strengthened the clarity, replicability and impact of the study. We hope that you will now consider this acceptable for publication in PLOS ONE.

Yours sincerely,

**Stefanie Bonfield, MSc**
PhD student, Centre for Cancer Screening, Prevention, and Early Diagnosis, Wolfson Institute of Population Health, Queen Mary University of London

**Dr Samantha Quaife, PhD CPsychol**

Reader in Behavioural Science, Centre for Cancer Screening, Prevention, and Early Diagnosis, Wolfson Institute of Population Health, Queen Mary University of London

| **No.** | **Comment** | **Response** |
| --- | --- | --- |
| **Reviewer#1** | | |
| **1.** | Dear Authors, The investigation of users’ preferences regarding cancer detection tests among people at risk of socioeconomic disadvantage can contribute to overcoming barriers to access those tests. Please see my comments below. When you respond to the comments, please provide your answers in a table with two columns, one with comments and the other with your answers. In the answer column, for each comment separately, include the revised text, in addition to your answer. | Thank you very much for your helpful comments, please see your responses laid out below as you request. Manuscript text is shown in blue font, and revisions are underlined. |
| **2.** | “Over 40% of individuals in the United Kingdom (UK) are diagnosed with cancer at an advance stage.(1)” Do you mean that 40% of the population is sick or that 40% of the diagnosed people are already in an advanced stage? Please revise the sentence to improve clarity. | Thank you, this has been revised for clarity:    *Over 40% of individuals with cancer in the United Kingdom (UK) are diagnosed at an advanced stage.(1)* |
| **3.** | Please correct the citation style. Insert the citation in the appropriate place: with the associated text, not after it (before “.” or “,”, not after them). | This has now been amended for all in-text citations. |
| **4.** | Please explain why it is important to know people’s preferences. It is important to motivate the usefulness of your study. Who cares about people’s preferences? From a supply-demand perspective, when the supply (tests) is much lower compared to the demand (need for tests), the supply side doesn’t care what the consumers (patients) want. If there are 100 pairs of shoes on the market and 1 million barefoot people needing shoes, who cares what color they like or even what size? Also, mention who should consider these preferences (scientists who create the tests, professionals who use it on patients, etc.). | Thank you, the benefit of preference research for those who receive tests as well as the healthcare systems involved in their delivery has been outlined in the introduction as follows:  *Preference research can guide test developers, policy makers and healthcare professionals in delivering early detection tests in ways that align with public and patient needs and optimise uptake. In turn this can reduce the burden of late diagnosis on patients and healthcare systems. This is important given that s*everal new test modalities to support early cancer detection are under development … |
| **5.** | Why did you focus only on people at risk of socioeconomic disadvantage? Do they represent the majority of sick people? What about the rest? Are they unimportant? | Thank you, the increased cancer risk and mortality facing these groups has been outlined in the first paragraph:  *Individuals experiencing poorer socioeconomic conditions make up a greater proportion of these groups who do not attend(3, 4), yet face greater incidence and lower survival rates across several types of cancer (5, 6).*  The reason for focusing on this group based on their under-representation in previous research is now reiterated in the final paragraph of the introduction:  *In addition to experiencing barriers to engaging with cancer tests, individuals experiencing socioeconomic deprivation also face additional challenges to taking part in health research.(20) Consequently, there have been calls to ensure that targeted efforts are made to include these groups in future research to narrow cancer disparities.(21, 22)* |
| **6.** | Can the results be extrapolated to people who are not at risk? Why/ Why not? | Thank you, we have now acknowledged the validity of extending the findings beyond the population in the discussion section for the first theme:  *It is likely that these findings extend to those who face similar barriers to participation regardless of socioeconomic disadvantage, underlining the value of achieving equitable access to improve uptake across the population.*  And the second theme:  *It is not clear from this study whether these perceptions are more prominent among those at risk of socioeconomic disadvantage or also persist across the population.* |
| **7.** | Please explain why the vignette task and think-aloud protocol are suitable for this study. | Thank you, the reasons for including the vignette task and think-aloud protocol have now been outlined in the ‘Interview Materials’ section:  *To help individuals consider which attributes of tests would be important, particularly those without previous experiences of being invited to take part in testing, two sets of interview materials were designed to introduce individuals to attributes of existing and emerging cancer detection tests. This included example test descriptions and a vignette task.* *Vignette tasks are a contemporary research tool that involve presenting participants with a hypothetical scenario to elicit individuals’ opinions and preferences.(36). It was expected that an individual’s first impressions and deliberations between test options would highlight which attributes were important for decision making and why, as well as which, if any, attributes were considered irrelevant or unimportant. For this reason, individuals were encouraged to read each set of materials and express their first impressions, thought processes and feelings out loud while engaging with each task, known as an adapted think-aloud protocol(36).* |
| **8.** | Please mention what data analysis methods you used and motivate your choice. | Thank you, this has been expanded in the Data analysis section as follows:  *The data were analysed in NVivo (version 12) using framework analysis and guided by seven recommended stages: Transcription, Familiarisation, Coding, Developing a working analytical framework, Applying the analytical framework, Charting the data into a framework matrix, Interpreting the data(41, 42).* *This method does not dictate whether data is analysed inductively or deductively and allows this to be informed by the research topic.(41) This was considered appropriate for systematically identifying similarities and differences in attitudes towards attributes of tests before interpreting thematic associations.(40-42) Other analytical approaches which are predominantly informed by the data (e.g. Grounded theory,) were not selected because the researchers were interested in exploring attitudes towards pre-defined attributes of cancer detection tests.* |
| **9.** | “This qualitative study aimed to use a vignette task and think…” The interview is not mentioned. Please add it. Also, it should be written that the study “used” instead of “aimed to use”. | Thank you for highlighting this, the sentence now reads:  *‘This qualitative interview study used a vignette task and think-aloud protocol to explore…’* |
| **10.** | “Qualitative semi-structured interviews supplemented with written descriptions of cancer detection tests, vignettes of tests scenarios and an adapted think-aloud protocol as participants were encouraged to deliberate between different test attributes” The predicate is missing. Please revise. | Thank you, this now reads as follows:  *‘Qualitative semi-structured interviews were conducted and supplemented with written descriptions of cancer detection tests, a vignette task (where hypothetical tests scenarios were presented) and an adapted think-aloud protocol (where participants were encouraged to deliberate between different test attributes while thinking out loud).’* |
| **11.** | “People living in London were recruited via community-based methods between 01/02/2023 – 08/02/2024.” It is not clear what the month and the day are. Please use words for the months. | This has been amended:  *‘People living in London were recruited via community-based methods between 1^st^ February 2023 and 1^st^ February 2024.’* |
| **12.** | Please briefly explain what a vignettes task and a think-aloud protocol are. | The ‘Study design and setting’ section now reads as:  *‘Qualitative semi-structured interviews were conducted and supplemented with written descriptions of cancer detection tests, a vignette task (where hypothetical tests scenarios were presented) and an adapted think-aloud protocol (where participants were encouraged to deliberate between different test attributes while thinking out loud).’*  This is further explained in the ‘Interview Materials’ section, see comment #7. |
| **13.** | Please explain what framework analysis is and why this was considered suitable and preferred to other methods for qualitative data. | Thank you, this has been expanded in the Data analysis section as follows:  *The data were analysed in NVivo (version 12) using framework analysis and guided by seven recommended stages: Transcription, Familiarisation, Coding, Developing a working analytical framework, Applying the analytical framework, Charting the data into a framework matrix, Interpreting the data(41, 42).* *This method does not dictate whether data is analysed inductively or deductively and allows this to be informed by the research topic.(41) This was considered appropriate for systematically identifying similarities and differences in attitudes towards attributes of tests before interpreting thematic associations.(40-42) Other analytical approaches which are predominantly informed by the data (e.g. Grounded theory,) were not selected because the researchers were interested in exploring attitudes towards pre-defined attributes of cancer detection tests.* |
| **14.** | “Participants were asked to read the descriptions of different types of early cancer detection tests and to think aloud while considering if anything about each test could be potentially off-putting from a patient perspective. Then participants were asked to engage with the vignette task and to think aloud while arranging the tests in order of preference that they thought the individual in the vignette would choose…. ” In addition to this description, please provide the interview script, the list with all questions used in the interview. Include the main questions, helping questions, etc. When presenting the questions, indicate the specific objective to which they respond. For example, it is mentioned that the study objective is to find out “how and why attributes of …. tests influence testing experiences and participation”. Show what questions focused on “how”, “why”, “experiences”, and “participation”. This information supports the replication of the study. | Thank you, the topic guide, including questions and prompts, has now been included in the supplementary file alongside the interview materials and instructions for the think-aloud protocol. We hope this improves the replicability of the study.  Questions were designed to be open-ended to allow individuals to reflect on the influence of attributes on their decisions to take part or their experiences of taking part, both of which were expected to be relevant to how and why attributes influence participation. This is now described in the interview materials section using examples to illustrate these objectives:  *A semi-structured topic guide was developed consisting of questions about individuals’ attitudes towards specific attributes of cancer detection tests. These related both to tests they had previous experience making decisions about (i.e., had been invited to consider before or had taken part in the test before) and/or cancer tests that might be offered to them in the future.* *Questions were designed to be open-ended (e.g. ‘What was your experience of being able to get to the test?’) to allow individuals to reflect on how attributes influenced their decision to test or their experiences (e.g. preferences for a test location) and/or why they held those views (e.g. preferred a local location because they were less mobile). Interview questions and prompts were loosely based on the COM-B model of behaviour change(7), to understand how and why attributes influenced their capability, opportunity and motivations to take part in testing. The subsequent analysis interpreted interviewees’ accounts to identify attributes that appeared to be instrumental in driving test decision-making and participation.* |
| **15.** | “FMW is also a qualified GP.” What does GP mean? | Thank you this has been clarified as a General Practitioner (GP). |
| **16.** | The text in Fig is very blurred. Please improve it. | Thank you, this has been addressed and the revised figure passes checks in PLOS’s NAAS tool and the PACE digital diagnostic tool. We believe the image may remain blurred in the PDF builder of the submission form, but is not when the image is downloaded. |
| **17.** | “Individuals discussed several attributes of early cancer detection tests that influence their capability and opportunity to take part based on their individual circumstances…..(the rest of the text)”. Vignettes are about imaginary people, Alex and Sam. The manuscript text presents the interview results as they were about the interview participants. This creates confusion. Please provide the necessary information to remove the confusion. How can you know they referred to themselves when they answered the questions about Alex and Sam and not to some imaginary people? What did you do to ensure that? | Thank you for raising this. We have now provided further information about the justification for including a third person vignette task in the ‘Interview materials’ section:  *Vignette tasks are appropriate for facilitating the discussion of sensitive topics.(36) It was expected that the inclusion of vignette scenarios described in the third person would allow individuals to share their preferences for attributes of tests without feeling obliged to share personal experiences or reasons for taking part in health testing if they did not wish to.*  We have also described the steps taken to ensure the results were reflective of participants’ personal preferences when discussing the vignette tasks in the ‘Data analysis’ section:  *Analysis of responses to the third person vignette tasks was focused on the reasons individuals cited for preferred attributes based on their own beliefs (e.g. ‘I think most people would prefer’) or personal circumstances (e.g. ‘If it was me ..’).* |
| **18.** | What do Q1, Q2, .. mean? Q from quote, or is this a code for each person? Please explain. | This has now been clarified in parentheses:  *Sub-themes are supplemented with numbered illustrative quotes (e.g. Quote 1 is denoted by Q1).* |
| **19.** | “Although, some highlighted that individuals who are less mobile may need the support from a relative (5).” Does “(5)” refer to the quote mentioned below this sentence? If yes, please write Q5. | Thank you for highlighting this error, this has now been amended to (Q5). |
| **20.** | The study objective is to find out “how and why attributes of …. tests influence testing experiences and participation”. Please present the results in a way that responds to the how and why, and highlights the experiences, and participation. | Thank you for raising this ambiguity. The reasons for analysing the influence of attributes on experiences and participation simultaneously has now been outlined in the ‘Data analysis’ section:  *The focus of the analysis was to explore which attributes contributed to negative experiences or attitudes towards taking part in testing and why. It was expected that the influence of attributes on experiences and participation would both be relevant to future engagement with tests. The COM-B model was used as a lens to explore the influence of attributes across experiences and participation*  In the ‘Thematic framework’ section, we have now reiterated the structure of the results in addressing the research objective:  *The first theme describes how and why test attributes were interpreted to prevent equitable access to early cancer detection tests by lowering individual’s capability and opportunity to take part. The second theme presents an interpretation of how and why test attributes influenced participants’ motivation to take part in testing.* |
| **Reviewer#2** | | |
| **21.** | Thank you for the opportunity to review this manuscript, which is timely regarding the likely future direction of cancer early detection, and in considering it from the perspective of those (inverse care law) least served by current cancer screening/early detection (or with the most to potentially benefit). | Thank you very much for reviewing the manuscript and providing helpful comments. We respond to each of these below in turn. |
| **22.** | It was great to see the involvement of public/patient members in the research material development - were they involved in any of the analysis or interpretation (I think from the coding description that they were not?). | Thank you for raising this. The PPI members were not involved in the analysis or interpretation of the findings as funding was not available to reimburse them for this part of the project. However, a copy of the findings was shared with them for reference. The following sentence has been added to the PPI section of the manuscript for clarification:  *‘They were not involved in the analysis or interpretation of the findings.’* |
| **23.** | Was any other health literacy lens used for the materials given the target audience? | Thank you. Additional considerations for those with lower literacy have been documented in the ‘Data collection’ section:  *[Sociodemographic survey] Participants were given a choice of whether to fill this in themselves or for the researcher to ask them the questions and record their responses.*  *During the vignette tasks, participants were told that they could look at one test option at a time if they preferred and did not have to examine all test options if they did not wish to.* |
| **24.** | Thank you for the COREQ this was also useful. | Thank you. |
| **25.** | I wasn't familiar with Think-Aloud Protocols - it would be useful if the description and a little more detail on this was provided at first use of the term. | Thank you for highlighting this, the first use of the term in the ‘Study design and setting’ section of the Methods section has now been adapted to clarify this:  *‘Qualitative semi-structured interviews were conducted and supplemented with written descriptions of cancer detection tests, a vignette task (where hypothetical tests scenarios were presented) and an adapted think-aloud protocol (where participants were encouraged to deliberate between different test attributes while thinking out loud).*  The topic guide including the instructions participants were given when asked to think aloud have also now been included as supplementary material to facilitate replication. |
| **26.** | It wasn't clear to me why participants needed a debrief sheet (line 164) - this is usually used in studies which involve deception? | Thank you, the research team felt it was appropriate to provide supporting information for participants in case there were any negative impacts of taking part in a potentially sensitive discussion. We have clarified the reason for this at the end of the ‘Data collection’ section:  *Participants were given a debrief sheet including a reminder of the aims of the research and contact details of support services in case they were negatively affected by the sensitive nature of the discussions or desired medical advice.* |
| **27.** | It wasn't clear to me why framework analyses was used over other types of qual analyses (line 168) - another sentence or two justification would be helpful to the reader. | This has been expanded in the Data analysis section as follows:  *This method does not dictate whether data is analysed inductively or deductively and allows this to be informed by the research topic.(41) This was considered appropriate for systematically identifying similarities and differences in attitudes towards attributes of tests before interpreting thematic associations.(40-42) Other analytical approaches which are predominantly informed by the data (e.g. Grounded theory,) were not selected because the researchers were interested in exploring attitudes towards pre-defined attributes of cancer detection tests.* |
| **28.** | Appropriate limitations were outlined including the nature of the sample being relatively highly educated, urban, and white/english speaking. | Thank you. |
| **29.** | I have two broad questions to the authors to consider whether any further clarification/detail in the manuscript may be useful:  The I-SAM (which includes COM-B) and CanTest are about informing/designing equitable screening/integrated programmes - from the protocol it appears the research wasn't originally designed using either framework - were these selected as part of the presentation of results? COM-B (the third part of I-SAM) appears to be the main aspect used in the results for this paper. Thinking about inequities, SES inequities as the focus of this work, the I-SAM proposes ways that theory and research can improve screening access in particular via patient and environmental factors - the background and the findings/discussion indicate a strong attention to the participant/patient factors but less on the opportunity side (particularly the environmental aspect/framing - opportunities to design programmes/approaches using these findings eg improving systems health literacy/information accessibility, how tests are offered and who by, ways to improve trustworthiness etc. | Thank you for raising this. Only the COM-B model was used to inform the design of the materials originally.  The CanTest framework outlines five research phases from cancer test development to implementation that are relevant to several stakeholders besides those receiving tests such as policy makers and test developers. Identifying the effects of tests on patients and inequalities is recommended within phases three and five of the framework, respectively. This study was therefore considered to be relevant within the framework from the perspective that the framework advocates and justifies the motivation for the study, rather than for the design and analysis of the study to be guided by it.  The I-SAM explores how capability, opportunity and motivation influence screening behaviour across factors including but not limited to attributes of the specific detection test such as mass media and community endorsement. The current study was interested specifically in how attributes of cancer tests in both screening and symptomatic contexts influence experiences and participation. The findings are therefore less focused on the communication and knowledge sharing aspects of testing because the aim was to understand how factors influenced by the type of test influenced participation. We have clarified the rationale for inclusion of the I-SAM in the ‘Data analysis’ section:  *The I-SAM was considered useful for informing how sub-themes should be arranged and labelled given its outline of factors influencing screening behaviour specifically, including but not limited to the attributes of tests.*  In the discussion section, we have made the implications for improving capability, opportunity and motivation more explicit*:*  *Emerging tests to support early cancer detection such as blood, breath and urine sample tests could improve individuals’ capability and opportunity to take part by requiring fewer time commitments (e.g. due to less travel required to locations, shorter procedures and potentially more varied appointment times), being available in more accessible locations and environments (e.g. community based settings), offering greater familiarity and privacy (e.g. not requiring individuals to remove clothing, although community settings may cause concerns about being seen by others), and imposing fewer physical and psychological demands (e.g. compared to physical and psychological discomfort associated with imaging, endoscopy and biopsy tests).*  *This suggests that the increased accessibility of sample tests for cancer detection may not lead to greater motivation to take part if individuals doubt the value of a test such as its importance or accuracy.* |
| **30.** | The second question was about including both screening (asymptomatic) and early detection (symptomatic) vignettes - do you think there was any confusion about this for the participants, and whether the symptomatic vignette impacted the perspectives/preferences more? Were the responses analyses separately for the different vignettes? | Thank you for raising this ambiguity. We know the public often confuse asymptomatic and symptomatic testing, and the topic guide was designed as carefully as possible to distinguish between these. We have now included the topic guide as a supplementary file which we hope clarifies the way that interview materials were introduced and the questions asked. More specifically, the topic guide included questions to understand how the specific vignette context influenced preferences, and prompts to understand differences between asymptomatic and symptomatic contexts where relevant or not mentioned by the participant. We believe this was an effective approach for understanding the influence of asymptomatic/symptomatic contexts on deliberations and decision making about test attributes. An example question and prompts used during the vignette task is shown below:  [Question] Are there any circumstances under which an attribute would be more/less important?  *PROMPT*: Would it matter why Sam/Alex was offered the test?  *PROMPT*: What about whether they felt well/unwell, how long they had been waiting for the appointment, whether they had had the test before?  The consideration of the vignette context has also been specified in the ‘Data analysis’ section:  *Analysis of responses to the third person vignette tasks was focused on the reasons individuals cited for preferred attributes based on their own beliefs (e.g. ‘I think most people would prefer’) or personal circumstances (e.g. ‘If it was me ..’).The analysis also considered the asymptomatic and symptomatic context of the vignette being discussed.* |
| **31.** | It was interesting that this study was about a broad range of test types across a broad set of potential screening/early detection settings - however one key note in the discussion (line 413) was that further research will be needed to understand perceived risk associated with tests in specific contexts. Given the potential nature of MCEDs/panels used for risk stratification/eligibility/screening/early detection do the authors think that studies in context will be possible? | Thank you for highlighting this, which is a really interesting point. We agree that this will depend on whether emerging biomarker tests are implemented for single or multiple types of cancer. We have now included this in the mentioned sentence:  *‘Further research will be needed to understand the perceived risk of a cancer diagnosis associated with sample tests used to rule out single and multiple types of cancer in specific contexts such as risk stratification and triage,* *and the most effective methods of communicating cancer risk information.’* |
